# Socioeconomic disparities and green space associated with myopia among Chinese school-aged students: A population-based cohort study

**DOI:** 10.7189/jogh.14.04140

**Published:** 2024-06-21

**Authors:** Congchao Lu, Yuyang Miao, Xuyang Yao, Zinuo Wang, Ruihua Wei, Bei Du, Yifan Liu, Jiayu He, Hongyue Sun, Yuanyuan Liu, Jing Yan, Guowei Huang, Xi Chen, Nai-jun Tang, Hua Yan

**Affiliations:** 1 Department of Occupational and Environmental Health, School of Public Health, Tianjin Medical University, Tianjin, China; 2 Tianjin Key Laboratory of Environment, Nutrition, and Public Health, Tianjin Medical University, Tianjin, China; 3 Department of Ophthalmology, Tianjin Medical University General Hospital, Tianjin, China; 4 Laboratory of Molecular Ophthalmology, Tianjin Medical University, Tianjin, China.; 5 Tianjin Key Laboratory of Ocular Trauma, Tianjin, China; 6 Tianjin Key Laboratory of Retinal Functions and Diseases, Tianjin Branch of National Clinical Research Center for Ocular Disease, Eye Institute and School of Optometry, Tianjin Medical University Eye Hospital, Tianjin, China; 7 School of Public Health, Tianjin Medical University, Tianjin, China; 8 School of Medicine, Nankai University, Tianjin, China

## Abstract

**Background:**

There is increasing evidence on the link between environmental factors and myopia in children and adolescents, yet with inconsistent conclusions. We investigated the associations between socioeconomic inequalities and green space with myopia in school-aged students participating in the Tianjin Child and Adolescent Research of Eye (TCARE) study.

**Methods:**

We obtained data from a population-based dynamic cohort study conducted in Tianjin, China, in 2021 and followed up in 2022. We included 1 245 271 participants from 16 districts with an average age of 11.6 years (standard deviation = 3.3) in our analysis. We synthesized their area-level SES through a prediction model that combined economic, educational, and health care variables and assessed the greenness levels surrounding the school using the Normalized Difference Vegetation Index (NDVI) based on data obtained through satellite remote sensing. We performed generalised linear mixed effects analyses for each myopia outcome separately, with adjustments for students’ sex, years of education completed, and the school’s geographical location.

**Results:**

We observed that students living in low SES areas had the highest prevalence of myopia (60.7%) in the last screening in 2022, as well as a higher incidence of one-year myopia (26.4%) compared to those residing in middle SES areas (22.7%). With a 0.1 increase in the 250, 500, and 1000 m buffer NDVI, the prevalence of myopia dropped by 6.3% (odds ratio (OR) = 0.937; 95% confidence interval (CI) = 0.915, 0.960), 7.7% (OR = 0.923; 95% CI = 0.900, 0.946), and 8.7% (OR = 0.913; 95% CI = 0.889, 0.937), respectively. The interaction analysis showed that low SES and low greenness exacerbate the prevalence of myopia. Findings from longitudinal analyses consistently demonstrated a correlation between higher values of NDVI and a slower progression of myopia. These findings remained robust across sensitivity analyses, including for variables on parental myopia and students’ behaviors.

**Conclusions:**

Exposure to green spaces could play a crucial role in slowing the progression of myopia among school-aged students. Myopia control policies should prioritise young populations residing in low SES areas with limited access to green spaces, as they face the highest potential risks.

Myopia presents a significant global public health concern, particularly due to its prevalence among school-aged children and adolescents [1,2]. It can adversely affect students’ academic performance and overall quality of life, while individuals with high myopia are at a higher risk of eye disorders such as retinal breaks and detachments [3]. Moreover, the condition is associated with a substantial economic burden both for society as a whole and for individuals, especially in countries with high myopia rates and for individuals with severe myopia [4]. In China, school screenings play a vital role in the detection of myopia among students. For example, data from the Chinese National Survey on Students’ Constitution and Health in 2019 indicated that the overall detection rate of myopia among Chinese Han children and adolescents aged 7–18 was 60.1% [5].

The high prevalence of myopia has been associated with various factors, including genetics, environmental factors, educational pressure, and lifestyle factors such as increased time spent near work and a lack of outdoor play [6]. Studies have also linked the development of myopia to a lack of exposure to natural light [7]. Moreover, time spent outdoors may be a modifiable factor in this correlation, with results from animal models and epidemiological studies lending credence to the hypothesis that natural light, particularly blue light with a broader spectrum of light waves, affects the eyes’ focusing and refractive ability. Moderate exposure to natural light could therefore help relax the eyes and enhance the ability to focus on distant objects [8]. Additionally, high levels of light exposure outdoors can promote the release of dopamine in the retina, inhibiting myopia development and serving as an ocular growth inhibitor [9].

Green spaces serve as excellent locations to increase exposure to natural light and reduce eye strain caused by near work. For example, during the coronavirus disease 2019 (COVID-19) pandemic, neighbourhood green spaces have become primary recreational and leisure areas for many Chinese residents due to travel restrictions [10]. Studies have shown that spending time in green spaces may protect against developing myopia [11,12]. Therefore, better understanding this association in the student population could contribute to future urban planning strategies to promote eye health. However, evidence from longitudinal cohort studies to support the protective effect of green spaces on myopia is limited [13].

A region’s socioeconomic status (SES) is closely associated with its population’s living environment, educational resources, and health care conditions. Therefore, it should be taken into account when considering the adverse effects of green space deprivation on myopia. For example, research has indicated that communities in districts with higher SES tend to have better access to green spaces, such as in the USA [14]. However, green spaces in China are often inconsistently distributed across different urban areas. For instance, high SES areas in Chinese urban cities often encounter challenges associated with inadequate green space due to land pressure and rapid urban expansion. Further, studies investigating the association between SES and health outcomes have had inconsistent results due to their use of different SES indicators [15–17]. In the rapidly developing Chinese society, establishing a supportive environment that improves the well-being of marginalised groups and minimises regional inequalities remains a key concern [18]. Therefore, relying solely on a single indicator, such as gross domestic product (GDP), is inadequate for effectively measuring a region’s overall development level [19,20].

Our primary aim was to investigate the influence of various regional SES levels and the exposure to green spaces around schools on the occurrence and progression of myopia among Chinese school students by using both cross-sectional and longitudinal data. Additionally, we wanted to explore the association between SES and green spaces in relation to myopia prevalence. We therefore hypothesised that school-aged students studying in schools with more green surroundings may experience a decreased risk of developing myopia compared to those residing in regions with lower SES levels and fewer green areas.

## METHODS

### Study design

The Tianjin Child and Adolescent Research of Eye (TCARE) study is a large-scale, population-based dynamic cohort study conducted in Tianjin, China. It relies on a long-term screening programme for myopia among students at primary and secondary schools, with annual follow-up. As part of this project, students received their own electronic health records, allowing them or their parents to check their screening results on the WeChat platform using smartphones for identity verification. The TCARE study aims to identify the determinants that contribute to the development of myopia and to better understand its progression in school-aged children and adolescents who are at risk of developing myopia. The study has received ethical approval from the Medical Ethics Committee of the Tianjin Medical University Eye Hospital and is conducted in accordance with the principles outlined in the Declaration of Helsinki.

### Participants

The study included students enrolled in primary schools, junior high schools, and senior high schools across 16 districts in Tianjin. Students who have previously undergone cataract surgery, laser refractive surgery or low-dose atropine were excluded from this study. The screening was additionally conducted for students in special education schools; however, we excluded their data from our analysis. Written informed consent was obtained from parents or guardians of the students participating in the study.

### Procedures

**Figure 1:**
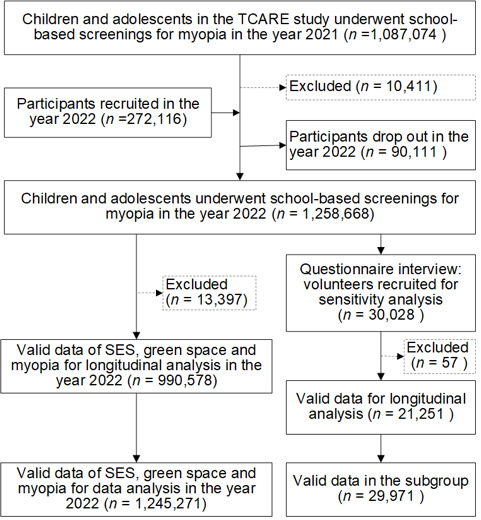
Flowchart of the participants in the TCARE study.

Between March 2021 and December 2021, the TCARE study enrolled 1 087 074 healthy Chinese school-aged students. Of these, 90 111 students with data in 2021 were not screened due to having graduated, while 272 116 students joined the study in 2022, yielding a total of 1 258 668 screened students in 2022 from 1476 schools (**Figure 1**). Trained health care professionals or school nurses performed myopia screening using a standardised protocol, which included visual acuity measurement and refractive examinations. The uncorrected visual acuity (UCVA) of each eye was measured at a distance of 5 meters using a standard logarithmic visual acuity E chart. Non-cycloplegic autorefraction procedures (Tianle RM-9600, Shanghai, China) involved spherical power, cylindrical power, and axis measurements. Each examination was performed three times for each eye of the students, and the average value was adopted. Further details were reported elsewhere [21].

Teachers collected demographic information (including age, sex, and educational information) before the screening. In October 2022, the TCARE Behaviors Questionnaire was distributed to a random sample online through the WeChat platform across eight districts in Tianjin. The questionnaires ascertained details regarding the family history of myopia as well as children’s behaviours (Table S1.1 in the **Online Supplementary Document**).

### Green space

We used the Normalized Difference Vegetation Index (NDVI) from the Moderate Resolution Imaging Spectrometer (MODIS) to assess the extent of green spaces within the school using remotely sensed data. The NDVI is a validated and internationally used parameter that describes the characteristics of surface vegetation, with values ranging from −1 to 1, with higher values indicating a higher concentration of vegetation around the school. We retrieved remote sensing images captured in the visible red and near-infrared bands from the MODIS sensor on board the Terra and Aqua polar-orbiting satellites, which are part of the US Earth Observing System. We calculated these images after radiometric calibration, atmospheric correction, and orthorectification (PRC parameters) according to the following equations:

NDVI = (NIR − RED) / (NIR + RED)

where NIR represents the near-infrared band and RED denotes the red band [22]. We reclassified negative values in the images as null values due to their common association with aquatic bodies. We then obtained school addresses from publicly available school addresses in the Tianjin education system and determined the average NDVI value for each plot within the buffer zone surrounding the school (250, 500, and 1000 meters) at intervals of 16 days. In most communities in China, the buffer zone within a one-kilometre radius requires approximately a 15-minute walking distance, which is also the maximum distance reachable by most urban residents on foot. We calculated the average NDVI from 2020 to 2022 over a three-year period to provide a more accurate assessment of changes in green space coverage. This approach helps reduce the impact of outliers or seasonal variability in single-year data and enhances the stability and reliability of the data.

### Area-level SES

We estimated the area-level SES through a prediction model that considered economic, educational, and health care variables to assess the district’s overall level of development [23]. Specifically, we used five predicted variables to create the area-level SES indicator: ‘regional GDP per capita,’ ‘percentage of educational budgetary expenditures in GDP,’ ‘percentage of health and wellness expenditures in GDP,’ ‘number of doctors and nurses per 1000 persons,’ and ‘number of beds in health care institutions per 1000 persons.’ We obtained these variables from the Tianjin Statistical Bureau’s 2021 annual data, published in the ‘2022 Tianjin Statistical Yearbook’ [24]. [Tianjin Statistical Bureau. 2022 Tianjin Statistical Yearbook. 2022. Available: https://stats.tj.gov.cn/nianjian/2022nj/zk/indexch.htm Accessed: 2 December 2022.], standardised and subjected the data for the principal component analysis using the following formula:

*Z’_ij_* = (*x_ij_* − *x̄*_j_) / *s_j_* (*i* = 1,2, … , *n*; *j* = 1,2, … , *m*)

After performing dimensionality reduction analysis, we combined them to create a composite indicator, denoted as *f*, and the evaluation function was formulated as follows:

*f* = *c_1_Z_1_* + *c_2_Z_2_* + … + *c_p_Z_p_*

Lastly, we categorised the 16 districts of Tianjin into high-level SES, middle-level SES, and low-level SES based on the sorted values of the composite indicator *f* (S2 in the **Online Supplementary Document**).

### Outcomes

Myopia was defined as spherical equivalent (SE) refraction of ≤ −0.50 dioptres (D) when UCVA was below 5.0 [25]. The SE was calculated by summing the sphere power with half of the cylinder power. The student was considered myopic if either eye was diagnosed with myopia during each screening.

Our primary outcome was the prevalence of myopia, defined as the proportion of students who were myopic during the final visit in 2022. The secondary outcome was the progression of myopia in this cohort during the one-year follow-up, including the incidence of myopia and the decline in both visual acuity levels and spherical equivalent refraction (SER). We defined the incidence of myopia as the proportion of students who did not have myopia at baseline, but developed it at the final visit; the decline in visual acuity levels as the change in UCVA; and the decline in SER was defined as the change in SER during the follow-up period.

### Statistical analysis

We presented demographic characteristics through means with standard deviations (SDs) for continuous variables and frequencies with percentages for categorical variables. We used the student *t*-test and χ^2^ test to compare these demographic characteristics between groups.

Further, we used a generalised linear mixed model to investigate the associations of myopia with area-level SES and green space around school, due to its ability to efficiently manage both fixed and random effects and to introduce interactions, thereby enhancing its explanatory and predictive power. This modelling approach helps address the non-independence of observations within identical groups, such as students within the same school. Due to the nested data structure, we considered schools as a random effects predictor, and the NDVI and other relevant confounders as fixed effects predictors. These confounders include variables such as the students’ sex, years of education completed [26], and the geographic area where the school was located. The model was estimated for each outcome separately. We then assessed the potential interaction of the SES and NDVI in relation to myopia in additive models, based on three indicators: relative excess risk due to interaction (RERI), attributable proportion (AP), and synergy index (SI) [27,28]. Finally, we performed sensitivity analyses to ensure the reliability of our findings, whereby we used logistic and linear regression analysis adjusted for potential factors. We did not impute missing data because only a small proportion (less than 1%) was missing.

We used R, version 4.2.2 (R Core Team, Vienna, Austria) and IBM SPSS, version 27.0 (IBM Corp, Armonk, New York, USA). All tests were conducted at α = 0.05.

## RESULTS

We included 1 245 271 school students in the analysis for 2022; 645 022 (51.8%) were boys and 600 249 (48.2%) were girls, with an overall average age of 11.6 years (SD = 3.3) (**Table 1**). The overall prevalence of myopia was 59%.

**Table 1:** Characteristics of the study population in Tianjin Child and Adolescent Research of Eye study in year 2022*

Characteristics	Table (n = 1 245 271)	Myopia (n = 734 325)	Non-myopia (n = 510 946)	*P*-value
Sex				<0.001
*Male*	645 022	363 933 (56.4)	281 089 (43.6)	
*Female*	600 249	370 392 (61.7)	229 857 (38.3)	
Age in years, x̄ (SD)	11.6 (3.3)	12.8 (3.0)	10.0 (2.8)	<0.001
Years of education completed, x̄ (SD)	5.6 (3.3)	6.8 (3.0)	3.9 (2.8)	<0.001
Level of education				<0.001
*Primary school students*	728 190	318 614 (43.8)	409 576 (56.2)	
*Junior high school students*	319 669	247 072 (77.3)	72 597 (22.7)	
*Senior high school students*	197 412	168 639 (85.4)	28 773 (14.6)	
Area-level socioeconomic status				<0.001
*Low*	413 804	251 145 (60.7)	162 659 (39.3)	
*Middle*	512 349	298 229 (58.2)	214 120 (41.8)	
*High*	319 118	184 951 (58.0)	134 167 (42.0)	
Geographic area,				<0.001
*Rural*	424 501	260 181 (61.3)	164 320 (38.7)	
*Suburban*	444 116	256 640 (57.8)	187 476 (42.2)	
*Urban*	376 654	217 504 (57.7)	159 150 (42.3)	
NDVI_250m_				<0.001
*Low (≤50th percentile)*	622 791	371 316 (59.6)	251 475 (40.4)	
*High (>50th percentile)*	622 480	363 009 (58.3)	259 471 (41.7)	
NDVI_500m_				<0.001
*Low (≤50th percentile)*	623,600	368 901 (59.2)	254 699 (40.8)	
*High (>50th percentile)*	621 671	365 424 (58.8)	256 247 (41.2)	
NDVI_1000m_				<0.001
*Low (≤50th percentile)*	622 649	372 194 (59.8)	250 455 (40.2)	
*High (>50th percentile)*	622 622	362 131 (58.2)	260 491 (41.8)	

NDVI – normalised difference vegetation index, SD – standard deviation, x̄ – mean

*Presented as n (%) unless specified otherwise. Differences among groups were tested using a *t* test or χ^2^ test.

**Figure 2:**
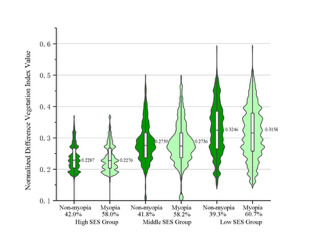
Differences in NDVI within 1000m buffer zones around the school between myopia and non-myopia groups with varying socio-economic statuses.

Stratified by SES, the low, middle, and high SES areas accounted for 33.2%, 41.1%, and 25.6% of the entire study population, respectively, with prevalence among these groups being 60.7%, 58.2%, and 58%. The NDVI values for the 250, 500, and 1000 metre buffer zones around the school were 0.265 (interquartile range (IQR) = 0.219, 0.332), 0.267 (IQR = 0.222, 0.328), and 0.272 (IQR = 0.228, 0.326), respectively. **Figure 2** shows the distribution of the various groups of green spaces. The differences in the prevalence of myopia among the various groups shown in **Table 1** were statistically significant according to univariate analysis (*P* < 0.05).

**Figure 3:**
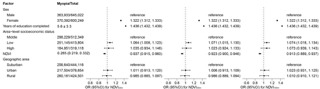
Characteristics and ORs with 95% CIs for NDVI at the 250-m, 500-m, and 1000-m buffer. CI – confidence interval, OR – odds ratio, NDVI – normalised difference vegetation index.

 The results indicated a higher prevalence of myopia in girls (odds ratio (OR) = 1.322; 95% confidence interval (CI) = 1.312, 1.333) and students with longer years of schooling (OR = 1.436; 95% CI = 1.432, 1.439). Moreover, students residing in low SES areas had a higher prevalence of myopia compared to their counterparts in middle SES districts. This correlation remained consistent across different models, including NVDI with various buffers, with ORs for SES ranging from 1.064 to 1.074. Furthermore, these findings suggest that with a 0.1 increase in the 250, 500, and 1000 metre buffer NDVI, the prevalence of myopia dropped by 6.3% (OR = 0.937; 95% CI = 0.915, 0.960), 7.7% (OR = 0.923; 95% CI = 0.900, 0.946), and 8.7% OR = 0.913; 95% CI = 0.889, 0.937), respectively. The prevalence of myopia was not associated with the geographic location of the school (**Figure 3**). In our separate analyses for boys and girls, we observed that boys in low SES areas had a higher prevalence of myopia compared to those residing in middle SES districts; however, this was not observed in girls (Tables S3.1.1 and S3.1.2 in the **Online Supplementary Document**). The correlation between NDVI and the prevalence of myopia remained robust for both groups across sensitivity analyses at different buffer distances.

For the additive model on the interaction between SES and NDVI, we categorised SES as high or middle vs low and dichotomised NDVI at the 50th percentile (**Table 2**). The reference group included areas with high to middle SES and high NDVI levels. This indicates a synergistic effect between SES and NDVI on myopia prevalence. Within the 250, 500, and 1000 metre buffer zones of green space, the RERI values were 0.108 (95% CI = 0.044, 0.172), 0.111 (95% CI = 0.046, 0.176), and 0.105 (95% CI = 0.037, 0.173) respectively. This shows that the relative excess risks from the additive interaction between low SES and low NDVI were 10.8%, 11.1%, and 10.5% for the 250, 500, and 1000 metre zones, respectively. Correspondingly, the joint effect has led to an increase in myopia risk by 9.6%, 9.7%, and 8.8%, respectively, as seen from the AP values. Moreover, the SI indicates that within a 1000 metre buffer zone, the additive interactions between low SES and low NDVI have led to a 2.108 higher myopia risk for participants with both low SES and low NDVI than for those exposed to only one risk factor (SI = 2.108; 95% CI = 1.165, 3.816).

**Table 2:** Myopia by additive interaction analysis of NDVI and area-level socioeconomic status*

	Area-level socioeconomic status				
**NDVI**	**High and middle**	**Low**	**RERI, aOR (95% CI)**	**Attributable proportion, aOR (95% CI)**	**Synergy index, aOR (95% CI)**
NDVI_250m_					
*High (>50th percentile)*	ref	1.088 (1.044, 1.134)	0.108 (0.044, 0.172)†	0.096 (0.042, 0.151)†	9.203 (0.110, 768.3)
*Low (≤50th percentile)*	0.925 (0.892, 0.960)	1.121 (1.061, 1.184)			
NDVI_500m_					
*High (>50th percentile)*	ref	1.094 (1.050, 1.140)	0.111 (0.046, 0.176)†	0.097 (0.043, 0.151)†	4.568 (0.744, 28.039)
*Low (≤50th percentile)*	0.937 (0.904, 0.971)	1.142 (1.080, 1.207)			
NDVI_1000m_					
*High (>50th percentile)*	ref	1.106 (1.062, 1.152)	0.105 (0.037, 0.173)†	0.088 (0.034, 0.142)†	2.108 (1.165, 3.816)†
*Low (≤50th percentile)*	0.989 (0.956, 1.023)	1.200 (1.137, 1.267)			

aOR – adjusted odds ratio, CI – confidence interval, NDVI – normalised difference vegetation index, ref – reference, RERI – relative excess risk due to interaction

*When additive interaction is absent, both RERI and attributable proportion (AP) are equal to 0 and synergy index (SI) is equal to 1. When additive interaction is synergistic effect, RERI or AP will be more than 0, or SI will be more than 1 (0 is clearly without the 95% CI of RERI and AP or 1 is clearly without the 95% CI of SI). When additive interaction is antagonism effect, RERI or AP will be less than 0, or SI will be less than 1 (0 is clearly without the 95% CI of RERI and AP or 1 is clearly without the 95% CI of SI).

†*P* < 0.05.

In the total sample, 990 578 students (79.5%) underwent a one-year follow-up between two screenings and were included in the longitudinal analysis. Out of the 446 641 students who did not have myopia during the first screening, 112 714 students were newly diagnosed with myopia in 2022, with an SER of −0.9 D (IQR = −1.4, −0.6). The one-year incidence of myopia was 25.2%. When considering myopia in both eyes (Table S3.2.1 in the **Online Supplementary Document**), both low (26.4%) and high (27.9%) SES areas had a higher incidence of myopia compared to the districts with middle SES (22.7%). However, when we performed separate analyses for the right and left eyes, we found that students from low SES areas had a higher incidence of myopia in their right eyes compared to those residing in districts with middle SES (Tables S3.2.2 in the **Online Supplementary Document**). A higher number of green spaces was associated with a reduced incidence of myopia. The ORs for NDVI at the buffer distances of 250, 500, and 1000 metre zones were 0.925 (95% CI = 0.893, 0.957), 0.914 (95% CI = 0.882, 0.948), and 0.903 (95% CI = 0.868, 0.939), respectively.

During the one-year follow-up period, we observed a decrease in the UCVA of both eyes among the participants (n = 990 578), from 4.85 (IQR = 4.50, 5.00) during the initial screening to 4.80 (IQR = 4.45, 5.00). Similarly, the SER also decreased from −0.9 D (IQR = −2.6, −0.1) to −1.2 D (IQR = −2.9, −0.3). In view of factors influencing the changes in UCVA, we saw that students residing in low SES areas experienced a slightly more rapid decline in UCVA compared to their counterparts in middle SES districts (Table S3.3 in the **Online Supplementary Document**). Conversely, an increase in NDVI_1000m_ showed a correlation with a slightly slower decline in UCVA (correlation coefficient *β* = 0.004; 95% CI = 0.000, 0.009). Regarding the factors influencing changes in SER, we found that students residing in both low and high SES areas experienced a rapid decline in SER compared to those in middle SES districts. However, there was no correlation between NDVI and the change in SER (Table S3.4 in the **Online Supplementary Document**).

We included 29 971 students who completed the questionnaires in the subgroups for the sensitivity analysis. The correlation between SES, green space, and the prevalence of myopia remained robust across all sensitivity analyses (Tables S4.1–4.4 in the **Online Supplementary Document**). Moreover, students who frequented green spaces more often had a lower prevalence of myopia, with ORs ranging from 0.921 to 0.922. Findings from the longitudinal analysis during the follow-up period (n = 21 251) confirmed the robustness of the findings regarding SES and the incidence of new myopia. Students residing in low SES areas exhibited a more rapid decline in UCVA, while those in high SES areas had a slower decline in UCVA, but a slightly rapid decline in SER. Higher greenness was correlated with a slower decline in UCVA (for NDVI_250m_, NDVI_500m_, and NDVI_1000m_) and SER (only for NDVI_250m_).

## DISCUSSION

We investigated the associations between socioeconomic disparities at the neighbourhood level, the availability of green spaces surrounding schools, and the prevalence of myopia among school-aged children and adolescents in a rapidly urbanising city in the Chinese mainland. Our results show that those students residing in low SES areas have a higher myopia prevalence than their peers residing in areas with moderate SES levels. Additionally, a greater number of green spaces was linked to a reduced myopia prevalence and a slower progression of this near-sighted condition.

Targeting and treating groups at high risk can effectively alleviate the future burden associated with myopia [29]. Our findings highlight the significance of comprehensive myopia health policies tailored to children and adolescents from diverse socioeconomic backgrounds, with the aim of promoting health equity during their critical developmental years. Over the past three decades, Tianjin has consistently had a higher prevalence of myopia among school students in China. At the national level, literature has previously found a correlation between the increasing prevalence of visual impairment among Chinese students and the rapid regional economic growth from 1985 to 2014 [30]. In certain economically disadvantaged provinces, these regions may encounter the challenge of rising myopia rates during economic development [31]. However, in economically developed provinces like Tianjin, considering the current active policies on myopia management by the Chinese government, the future risk of myopia prevalence may be concentrated in areas within the region with relatively lower SES levels, with a focus on boys, since they are at greater risk of myopia. Limited health care and education funding may result in individuals in low socioeconomic areas facing barriers to accessing eye health care, thereby increasing the risk of myopia development. When determining the classification of regions as underdeveloped areas, the government should consider not only the sole indicator of SES such as GDP, but rather a comprehensive assessment of economic, educational, and health care conditions. However, the economic development and shifts in government investments in education and health care across various regions may result in alterations to regional SES. Future research is required to determine the long-term effects of these changes in SES on the development of myopia.

Furthermore, we identified a synergistic association between the coexistence of low SES and limited green spaces, resulting in an additional increased risk of myopia. As of yet, this association has not been sufficiently researched. There is a need for increased allocation of health care resources for vision care to mitigate potential increases in pathological and high myopia cases in these deprived regions. Our results show that public policy should prioritise disadvantaged young populations with less access to green space as they possess the highest potential [32].

Previous research has suggested a need for more substantial evidence regarding the association between green space exposure and myopia, primarily due to the identification of cross-sectional correlations [11,12,33]. Our study addresses this based on a large cohort of over a million students, but also through a subgroup analysis of about thirty thousand individuals and their genetic and behavioural factors. Based on this, we can hypothesise that a greater presence of green spaces surrounding educational institutions is linked to a slower progression of myopia, as indicated by one-year changes of UCVA and SER; however, we did not find it to be linked to the occurrence of new myopia cases. The literature indicates that a high NDVI does not necessarily correlate with increased usage of green spaces [34]. Alternatively, we could argue that incorporating the frequency of students’ actual visits to green spaces into the study might capture essential details about their interactions with these areas. Consequently, the importance of NDVI, derived from remote sensing data, is reduced compared to direct behavioural measurements, thereby diminishing its independent predictive capability in statistical terms. Further research is necessary to determine whether establishing more green spaces can effectively reduce the incidence of myopia among children and adolescents, while considering multiple factors.

We likewise found that increasing the buffer zone of green spaces enhances their protective effect, with our interaction analysis indicating that, within larger buffer zones (e.g. 1000 m), the combined effect of low SES and limited green space produces more robust statistical outcomes compared to those in 250 and 500 metre buffer zones. This effect can be attributed to including more green spaces within the buffer zone, providing students with increased exposure to natural light and reducing the proximity of near work activities. While existing research has not yet provided sufficient evidence to definitively determine the optimal extent of expanding green spaces around schools, it may be more critical at present to focus on enhancing their accessibility. Therefore, urban planners should pay special attention to easily accessible small green spaces in residential neighbourhoods, such as pocket parks, especially in densely populated but economically underdeveloped areas.

Our study also provides supporting evidence that educational pressures and excessive near work, such as inappropriate reading distance and spending more time using screens, on the heightened prevalence of myopia. Based on our findings, we can suggest that establishing healthy behavioural habits is crucial in reducing the risk of myopia progression, particularly for children and adolescents who have increased their screen time during the COVID-19 pandemic [35,36].

In comparison to similar research, the strength of this study is its larger sample size achieved through a school-based screening for national myopia control. Additionally, we derived the SES index used here from publicly available indicators in the regional statistical yearbook, which is accessible online, making it useful for establishing comparable socioeconomic indicators across different regions. However, some limitations have to be considered. First, the measurement of refractive errors relied on non-cycloplegic refraction, although this method is suitable and cost-effective for myopia screening purposes. However, noncycloplegic refractions may overestimate myopia in young children due to incomplete relaxation of the ciliary muscle, leading to inaccurate measurements of refractive error, especially in individuals with strong accommodative ability at a young age. Second, although NDVI is commonly used to measure greenness in health research, it is based on remote sensing data and may not fully capture the perceived greenness and three-dimensionality experienced by human vision in real-world environments [37]. Third, our study population was from Tianjin, which is one of the four municipalities in China with a higher economic level, making it less generalisable to the rest of the country. Finally, due to limitations of our study design, further investigations with longer follow-up durations are necessary to validate these relationships.

## CONCLUSIONS

Our findings suggest that there is an association between area-level SES, the availability of green spaces surrounding schools, and myopia in school students. However, the development of myopia is influenced by various factors, so longitudinal studies are necessary to better understand these relationships and establish causality. Future studies should track the long-term effects of exposure to various types and qualities of green spaces on myopia progression in children and adolescents. Such evidence would support global strategies to reduce the growing epidemic of myopia among children and adolescents.

## Additional material


Online Supplementary Document
