# Supplementary material for: Socioeconomic disparities and green space associated with myopia among Chinese school-aged students: A population-based cohort study
Source: J Glob Health. 2024 Jun 21;14:04140. doi: 10.7189/jogh.14.04140 (PMC11187523; doi:10.7189/jogh.14.04140)
Supplement: Online Supplementary Document [file jogh-14-04140-s001.pdf]

Supplementary appendix

S1. Description of the questions regarding parental myopia and children's behaviors used in this study. (Page 2)

Table S1.1: Description of questions used in this study derived from the TCARE behaviors questionnaire. **(Page 3)**

S2. Instruction to derive the area-level socioeconomic status. (Page 5)

Table S2.1: Description of variables used for create the area-level socio-economic status indicator. **(Page 6)**

Table S2.2: Variables in the prediction model of socio-economic status by principal component analysis. **(Page 7)**

Figure S2.1. Colour-coded diagram of the socio-economic status of Tianjin districts. **(Page 8)**

S3. Supporting tables and figure for the supplementary results in this study. (Page 9)

Table S3.1.1: Characteristics of myopia and non-myopia among 645,022 boys in the Tianjin Child and Adolescent Research of Eye study. **(Page 10)**

Table S3.1.2: Characteristics of myopia and non-myopia among 600,249 girls in the Tianjin Child and Adolescent Research of Eye study. **(Page 11)**

Table S3.2.1: Characteristics of new myopia and non-myopic among 446,641 students during one year follow-up in the Tianjin Child and Adolescent Research of Eye study. **(Page 12)**

Table S3.2.2: Characteristics of new myopia and non-myopic in right eyes among 503,225 students during one year follow-up in the Tianjin Child and Adolescent Research of Eye study. **(Page 13)**

Table S3.2.3: Characteristics of new myopia and non-myopic in left eyes among 522,090 students during one year follow-up in the Tianjin Child and Adolescent Research of Eye study. **(Page 14)**

Table S3.3: The estimated regression coefficients of changes in uncorrected visual acuity for both eyes among 990,578 students according to green space exposure in different buffers. **(Page 15)**

Table S3.4: The estimated regression coefficients of changes in sphere equivalent for both eyes among 990,578 students according to green space exposure in different buffers. **(Page 16)**

S4. Supporting tables for the results of sensitivity analysis in this study. (Page 17)

Table S4.1: Results of the sensitivity analysis of factors associated with myopia among 29, 971 students in the TCARE study. **(Page 18)**

Table S4.2: Odds ratios (ORs) of new myopia among 10,652 students associated with socio-economic statuses and green space exposure. **(Page 19)**

Table S4.3: The estimated regression coefficients of changes in uncorrected visual acuity for both eyes among 21,251 students according to green space exposure in different buffers. **(Page 20)**

Table S4.4: The estimated regression coefficients of changes in sphere equivalent for both eyes among 21,251 students according to green space exposure in different buffers. **(Page 21)**

S1. Description of the questions regarding parental myopia and children's behaviors used in this study.

Parents provided information about parental myopia and their children's behaviors through an electronic questionnaire. Questions included outdoor activities, such as the time spent playing outdoors and the frequency of going to green spaces. Additionally, near-work-related behaviors were assessed, including screen time and reading distance. Exposure to light in the home environment encompasses the types of lamps used for near work and sleeping with night lights. Dietary preferences, such as salt intake, frequency of snack intake, frequency of seafood intake, and frequency of soft drinks, were also recorded. A description of these questions is presented in Table S1.1.

Table S1.1: Description of questions used in this study derived from the TCARE behaviors questionnaire.

Concept	Questions	Options
Parental myopia	* Is the child's mother myopic?	Yes / No
	* Is the child's father myopic?	Yes / No
Outdoor play	* What is the total number of hours you spend outdoors (including any activity, not limited to sports) each day?	<1h<br>1-2h<br>2-3h<br>≥3h
Frequency of going to green spaces	* How often does the child visit a park, forest, or other green spaces?	Almost every day<br>About once a month<br>2-3 times per month<br>Once every few months<br>Never
Screen time	* The average amount of time you spend using electronics (including cell phones, TV, computers, etc.) each day?	___ hours
Reading distance	* Do you read at a distance greater than one foot (33cm) in your daily life?	Yes / No
Types of lamps for near work	* What type of desk lamp do you use for close reading and writing?	Incandescent lamps<br>Fluorescent lamps<br>LED lamps<br>Almost no use of desk lamps
Sleeping with night lights	* Do you sleep with night lights at night?	Never<br><br>Sometimes
Dietary salt intake	* What is the salt content of your child's diet?	Less than 4g/day (lighter taste)<br>4-6g/day (normal taste)<br>Above 6 g/day (salty taste)
Frequency of snack intake	* What is the frequency of your child's intake of snacks and sweetbreads (snacks, desserts, cakes, etc.)?	Hardly ever<br>Less than 1 time/week<br>2-3 times/week<br>4-6 times/week<br>1 time/day<br>2 or more times/day
Frequency of seafood intake	* What is the frequency of your child's intake of fish, shrimp, crab, shellfish, or other aquatic products?	Hardly ever<br>Less than 1 time/week<br>2-3 times/week<br>4-6 times/week

Concept	Questions	Options
Frequency of soft drinks	* What is the frequency of your child's intake of carbonated beverages (sodas, colas, etc.) and milk tea?	1 time/day<br>2 or more times/day<br>Hardly ever<br>Less than 1 time/week<br>2-3 times/week<br>4-6 times/week<br>1 time/day<br>2 or more times/day

S2. Instruction to derive the area-level socioeconomic status.

The area-level SES in this study was derived by a prediction model combining economic, educational, and healthcare variables to estimate the level of development of a district. The data were derived from the Tianjin Statistical Bureau's 2021 annual data, as published in the "2022 Tianjin Statistical Yearbook". From this yearbook, we gathered regional data including population, the gross domestic product (GDP) of each district (in hundreds of millions of yuan), educational budgetary expenditures (in hundreds of millions of yuan), health and wellness expenditures (in hundreds of millions of yuan), the number of licensed (assistant) doctors per 1,000 people, the number of registered nurses per 1,000 people, and the number of beds in healthcare institutions per 1,000 people. Using this data, four additional variables were generated: 'regional GDP per capita', 'percentage of educational budgetary expenditures in GDP', 'percentage of health and wellness expenditures in GDP', and 'number of doctors and nurses per 1,000 persons'. These variables, along with the 'number of beds in healthcare institutions per 1,000 persons', were utilized to create the area-level SES indicator for this study. Table S2.1 presents the data derived from the '2022 Tianjin Statistical Yearbook(https://stats.tj.gov.cn/nianjian/2022nj/zk/indexch.htm)'.

These data were standardized and subjected to principal component analysis. The Z-values of each indicators for each district after standardization are shown in Table S2.2, Five standardized variables were entered into the principal component analysis: 'regional GDP per capita', 'number of beds in healthcare institutions per 1,000 persons', 'number of doctors and nurses per 1,000 persons', 'percentage of educational budgetary expenditures in GDP', and 'percentage of health and wellness expenditures in GDP' The following formula was used:

$$Z'_{ij} = \left(x_{ij} - \overline{x_j}\right) / s_j (i = 1,2, \ldots, \mathrm{n}; j = 1,2, \ldots, \mathrm{m})$$

The correlation matrix for each indicator was calculated using standardized data, followed by eigenvalue decomposition. The first two principal components were then selected based on the magnitudes of the eigenvalues to explain the majority of the variance. After the dimensionality reduction analysis, the data were combined into a composite indicator f, and the evaluation function was constructed as follows:

$$f = c_1 Z_1 + c_2 Z_2 + \cdots + c_p Z_p$$

The Table S2.2 shows a summary of the results of the principal component analysis.

Based on the sorted values of f, the 16 districts of Tianjin were categorized into high-level SES (Heping, Hexi, Hebei, Hongqiao, and Nankai), middle-level SES (Binhai new district, Jinnan, Beichen, Hedong, Baodi, and Xiqing), and low-level SES (Dongli, Ninghe, Jizhou, Jinghai, and Wuqing). See Figure S2.1.

Table S2.1: Description of variables used for create the area-level socio-economic status indicator.

District	Regional GDP per capita (10,000 CNY)	Number of beds in healthcare institutions per 1,000 persons	Number of doctors and nurses per 1,000 persons	Percentage of educational budgetary expenditures in GDP (%)	Percentage of health and wellness expenditures in GDP (%)
Heping	13.31	13.29	22.27	1.94	0.48
Hexi	13.54	11.16	14.34	1.85	0.67
Nankai	11.29	10.38	14.88	2.39	0.91
Hebei	14.67	6.19	9.15	3.47	1.16
Hongqiao	13.34	8.69	9.36	6.78	2.20
Binhai new district	13.66	4.32	7.03	1.24	0.38
Jinnan	12.17	4.52	6.87	4.53	1.46
Beichen	11.64	4.9	7.19	2.36	1.07
Hedong	10.71	4.03	6.51	4.11	1.46
Baodi	10.94	3.71	4.24	5.65	1.99
Xiqing	11.84	2.24	3.25	2.08	0.97
Jizhou	10.39	2.56	4.49	10.00	3.31
Ninghe	10.08	3.82	4.13	3.70	1.51
Dongli	11.11	2.09	3.11	1.94	0.87
Jinghai	10.40	2.45	3.66	4.01	1.54
Wuqing	9.36	3.6	4.37	3.32	1.52

Table S2.2: Variables in the prediction model of socio-economic status by principal component analysis.

District	Z-score of X_1	Z-score of X_2	Z-score of X_3	Z-score of X_4	Z-score of X_5	Coefficients 1	Coefficients 2	*f*
Heping	1.01	2.24	2.74	-0.79	-1.18	-0.84	2.84	1.85
Hexi	1.15	1.63	1.24	-0.83	-0.92	-1.30	1.83	1.23
Nankai	-0.32	1.40	1.34	-0.59	-0.59	-0.27	1.10	0.75
Hebei	1.89	0.20	0.25	-0.11	-0.25	-0.99	1.05	0.66
Hongqiao	1.02	0.92	0.29	1.36	1.17	-0.08	0.69	0.64
Binhai new district	1.23	-0.34	-0.15	-1.10	-1.33	-1.13	0.67	0.45
Jinnan	0.25	-0.28	-0.18	0.36	0.16	0.13	-0.03	-0.05
Beichen	-0.09	-0.17	-0.12	-0.60	-0.38	-0.25	-0.16	-0.14
Hedong	-0.70	-0.42	-0.24	0.18	0.15	0.55	-0.56	-0.44
Baodi	-0.55	-0.51	-0.67	0.86	0.89	0.71	-0.91	-0.54
Xiqing	0.04	-0.94	-0.86	-0.72	-0.51	-0.34	-0.68	-0.57
Jizhou	-0.91	-0.84	-0.63	2.79	2.71	2.16	-1.49	-0.73
Ninghe	-1.11	-0.48	-0.70	0.00	0.23	0.49	-1.07	-0.74
Dongli	-0.44	-0.98	-0.89	-0.79	-0.65	-0.13	-0.84	-0.76
Jinghai	-0.90	-0.87	-0.78	0.13	0.27	0.62	-1.14	-0.81
Wuqing	-1.58	-0.54	-0.65	-0.18	0.24	0.68	-1.33	-0.81

X_1, regional GDP per capita; X_2, number of beds in healthcare institutions per 1,000 persons; X_3, number of doctors and nurses per 1,000 persons; X_4, percentage of educational budgetary expenditures in GDP; X_5, percentage of health and wellness expenditures in GDP.

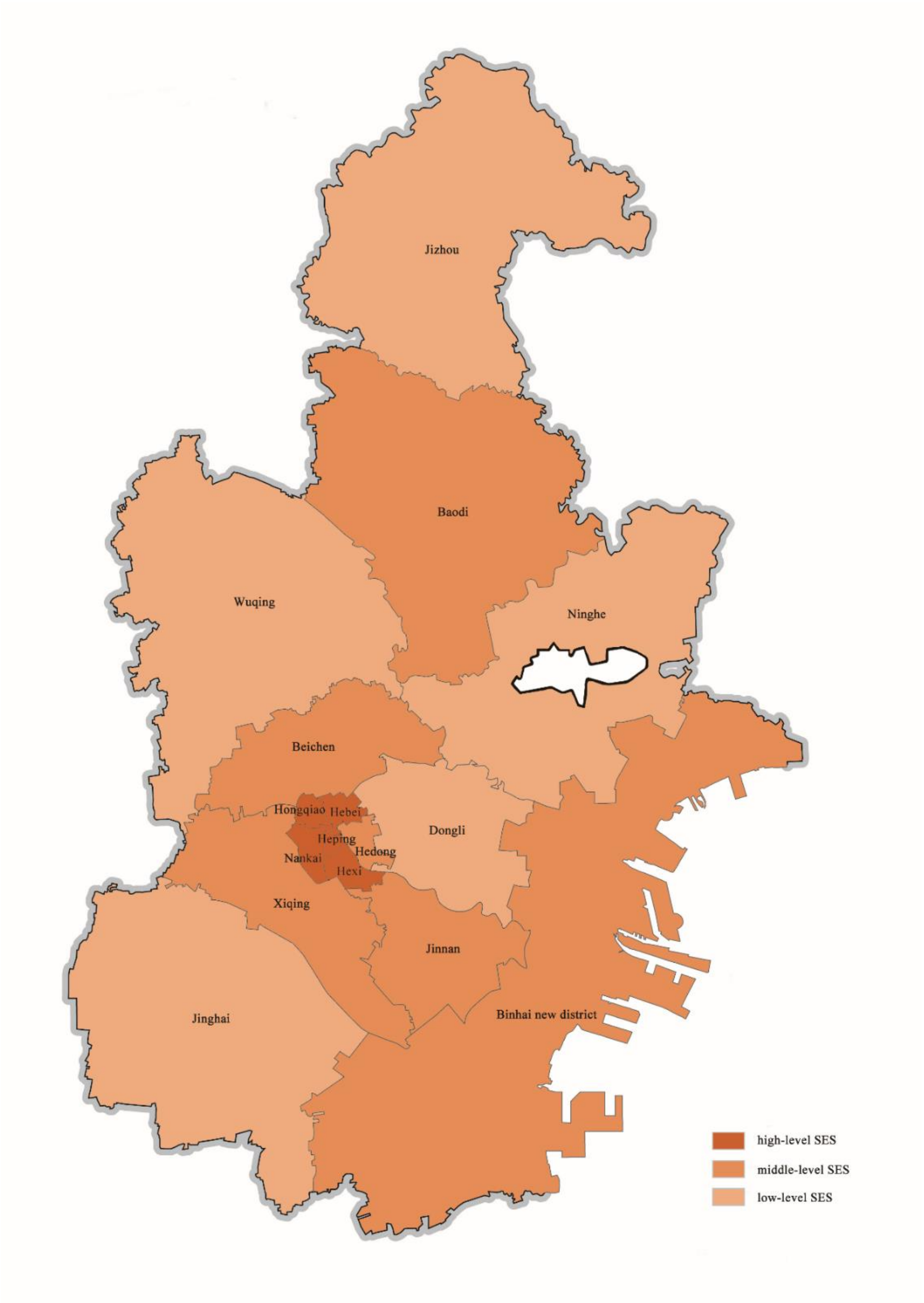

Figure S2.1. Colour-coded diagram of the socio-economic status of Tianjin districts.

S3. Supporting tables and figure for the supplementary results in this study.

Table S3.1.1: Characteristics of myopia and non-myopia among 645,022 boys in the Tianjin Child and Adolescent Research of Eye study.

Table S3.1.2: Characteristics of myopia and non-myopia among 600,249 girls in the Tianjin Child and Adolescent Research of Eye study.

Table S3.2.1: Characteristics of new myopia and non-myopic among 446,641 students during one year follow-up in the Tianjin Child and Adolescent Research of Eye study.

Table S3.2.2: Characteristics of new myopia and non-myopic in right eyes among 503,225 students during one year follow-up in the Tianjin Child and Adolescent Research of Eye study.

Table S3.2.3: Characteristics of new myopia and non-myopic in left eyes among 522,090 students during one year follow-up in the Tianjin Child and Adolescent Research of Eye study.

Table S3.3: The estimated regression coefficients of changes in uncorrected visual acuity for both eyes among 990,578 students according to green space exposure in different buffers.

Table S3.4: The estimated regression coefficients of changes in sphere equivalent for both eyes among 990,578 students according to green space exposure in different buffers.

Table S3.1.1: Characteristics of myopia and non-myopia among 645,022 boys in the Tianjin Child and Adolescent Research of Eye study.

Characteristics	Total (N = 645,022)	Myopia (n = 363,933)	Non-myopia (n = 281,089)	Model 1 $NDVI_{250m}$ included	Model 2 $NDVI_{500m}$ included	Model 3 $NDVI_{1000m}$ included
Normalized difference vegetation index (NDVI), P50 (P25, P75)	0.264 (0.219, 0.329)	0.263 (0.218, 0.327)	0.265 (0.220, 0.332)	**0.917 (0.895, 0.939)**	**0.903 (0.881, 0.926)**	**0.890 (0.867, 0.914)**
Years of education completed, mean (SD)	5.6 (3.2)	6.8 (3.0)	4.1 (2.9)	**1.379 (1.375, 1.384)**	**1.379 (1.375, 1.384)**	**1.379 (1.375, 1.383)**
Area-level socioeconomic status, n (%)						
Middle	269,115	149,240 (55.5)	119,875 (44.5)	Reference	Reference	Reference
Low	164,478	92,274 (56.1)	72,204 (43.9)	**1.083 (1.028, 1.140)**	**1.090 (1.036, 1.147)**	**1.095 (1.039, 1.153)**
High	211,429	122,419 (57.9)	89,010 (42.1)	1.049(0.941,1.169)	1.034(0.932,1.148)	1.050 (0.941, 1.172)
Geographic area, n (%)						
Suburban	232,694	128,458 (55.2)	104,236 (44.8)	Reference	Reference	Reference
Rural	217,825	126,951 (58.3)	90,874 (41.7)	0.944 (0.844, 1.055)	0.941 (0.848, 1.045)	0.971 (0.868, 1.086)
Urban	194,503	108,524 (55.8)	85,979 (44.2)	0.999 (0.900, 1.109)	0.991 (0.898, 1.095)	1.013 (0.912, 1.126)

Bold: $P < 0.05$.

Table S3.1.2: Characteristics of myopia and non-myopia among 600,249 girls in the Tianjin Child and Adolescent Research of Eye study.

Characteristics	Total (N = 600,249)	Myopia (n = 370,392)	Non-myopia (n = 229,857)	Model 1 $NDVI_{250m}$ included	Model 2 $NDVI_{500m}$ included	Model 3 $NDVI_{1000m}$ included
Normalized difference vegetation index (NDVI), P50 (P25, P75)	0.266 (0.220, 0.333)	0.266 (0.219, 0.333)	0.266 (0.220, 0.332)	**0.964 (0.937, 0.991)**	**0.950 (0.923, 0.979)**	**0.945 (0.915, 0.975)**
Years of education completed, mean (SD)	5.7 (3.3)	6.8 (3.0)	3.8 (2.8)	**1.379 (1.375, 1.384)**	**1.379 (1.375, 1.384)**	**1.379 (1.375, 1.383)**
Area-level socioeconomic status, n (%)						
Middle	243,234	148,989 (61.3)	94,245 (38.7)	Reference	Reference	Reference
Low	202,375	128,726 (63.6)	73,649 (36.4)	1.044 (0.982,1.11)	1.05 (0.987, 1.116)	1.051 (0.989, 1.118)
High	154,640	92,677 (59.9)	61,963 (40.1)	1.005 (0.879,1.149)	0.998 (0.875, 1.14)	1.006 (0.887, 1.141)
Geographic area, n (%)						
Suburban	211,422	128,182 (60.6)	83,240 (39.4)	Reference	Reference	Reference
Rural	206,676	133,230 (64.5)	73,446 (35.5)	1.029 (0.898, 1.178)	1.034 (0.904, 1.182)	1.049 (0.923, 1.192)
Urban	182,151	109,980 (60.4)	73,171 (39.3)	1.022 (0.899, 1.162)	1.021 (0.899, 1.159)	1.031 (0.914, 1.165)

Bold: $P < 0.05$.

Table S3.2.1: Characteristics of new myopia and non-myopic among 446,641 students during one year follow-up in the Tianjin Child and Adolescent Research of Eye study.

Characteristics	Total (n = 446,641)	Myopia (n = 112,714)	Non-myopia (n = 333,927)	Model 1 NDVI$_{250m}$ included	Model 2 NDVI$_{500m}$ included	Model 3 NDVI$_{1000m}$ included
Normalized difference vegetation index (NDVI), P50 (P25, P75)	0.269 (0.223, 0.334)	0.264 (0.219, 0.330)	0.270 (0.225, 0.336)	**0.925 (0.929, 0.972)**	**0.914 (0.882, 0.948)**	**0.903 (0.868, 0.939)**
Sex, n (%)						
Male	244,296	57,563 (23.6)	186,733 (76.4)	Reference	Reference	Reference
Female	202,345	55,151 (27.3)	147,194 (72.7)	**1.281 (1.263, 1.299)**	**1.281 (1.263, 1.299)**	**1.281 (1.263, 1.299)**
Years of education completed, mean (SD)	4.3 (2.5)	4.7 (2.5)	4.2 (2.5)	**1.144 (1.138, 1.149)**	**1.144 (1.138, 1.149)**	**1.143 (1.138, 1.149)**
Area-level socioeconomic status, n (%)						
Middle	184,565	41,841 (22.7)	142,724 (77.3)	Reference	Reference	Reference
Low	148,853	39,251 (26.4)	109,602 (73.6)	**1.291 (1.198, 1.391)**	**1.298 (1.205, 1.399)**	**1.303 (1.209, 1.404)**
High	113,223	31,622 (27.9)	81,601 (72.1)	**1.294 (1.087, 1.541)**	**1.295 (1.088, 1.540)**	**1.290 (1.086, 1.532)**
Geographic area, n (%)						
Suburban	168,780	39,515 (23.4)	129,265 (76.6)	Reference	Reference	Reference
Rural	147,104	37,759 (25.7)	109,345 (74.3)	1.021 (0.944, 1.105)	1.025 (0.948, 1.108)	1.033 (0.955, 1.118)
Urban	130,757	35,440 (27.1)	95,317 (72.9)	0.940 (0.794, 1.112)	0.934 (0.789, 1.105)	0.932 (0.789, 1.102)

Bold: $P < 0.05$.

Table S3.2.2: Characteristics of new myopia and non-myopic in right eyes among 503,225 students during one year follow-up in the Tianjin Child and Adolescent Research of Eye study.

Characteristics	Total (n=503,225)	Myopia (n=154,726)	Non-myopia (n=348,499)	Model 1 $NDVI_{250m}$ included	Model 2 $NDVI_{500m}$ included	Model 3 $NDVI_{1000m}$ included
Normalized difference vegetation index (NDVI), P50 (P25, P75)	0.269 (0.223, 0.334)	0.265 (0.219, 0.330)	0.270 (0.225, 0.336)	**0.919 (0.878, 0.961)**	**0.906 (0.865, 0.949)**	**0.895 (0.851, 0.941)**
Sex, n (%)						
Male	273,562	78,925 (28.9)	194,627 (71.1)	Reference	Reference	Reference
Female	229,663	75,791 (33.0)	153,872 (67.0)	**1.073 (1.049, 1.097)**	**1.073 (1.049, 1.097)**	**1.073 (1.049, 1.097)**
Years of education completed, mean (SD)	4.5(2.6)	5.1 (2.6)	4.2 (2.5)	**1.258 (1.249, 1.268)**	**1.258 (1.249, 1.268)**	**1.258 (1.249, 1.268)**
Area-level socioeconomic status, n (%)						
Middle	215,150	65,677 (30.5)	149,473 (69.5)	Reference	Reference	Reference
Low	175,358	60,622 (34.6)	85,425 (65.4)	**1.229 (1.114, 1.355)**	**1.237 (1.122, 1.364)**	**1.240 (1.125, 1.367)**
High	131,582	46,157 (35.1)	85,425 (64.9)	0.935 (0.746, 1.173)	0.936 (0.751, 1.167)	0.932 (0.747, 1.163)
Geographic area, n (%)						
Suburban	152,163	52,320 (34.4)	99,843 (65.6)	Reference	Reference	Reference
Rural	173,890	59,033 (33.9)	114,857 (66.1)	**0.865 (0.780, 0.959)**	**0.869 (0.784, 0.962)**	**0.876 (0.791, 0.971)**
Urban	196,037	61,103 (31.2)	134,934 (68.8)	0.996 (0.800, 1.240)	0.989 (0.800, 1.223)	0.988 (0.797, 1.224)

Bold: $P < 0.05$.

Table S3.2.3: Characteristics of new myopia and non-myopic in left eyes among 522,090 students during one year follow-up in the Tianjin Child and Adolescent Research of Eye study.

Characteristics	Total (n=522,090)	Myopia (n=172,456)	Non-myopia (n=349,634)	Model 1 NDVI$_{250m}$ included	Model 2 NDVI$_{500m}$ included	Model 3 NDVI$_{1000m}$ included
Normalized difference vegetation index (NDVI), P50 (P25, P75)	0.268 (0.223, 0.334)	0.265 (0.219, 0.330)	0.270 (0.225, 0.336)	**0.922 (0.895, 0.949)**	**0.911 (0.884, 0.940)**	**0.898 (0.869, 0.928)**
Sex, n (%)						
Male	283,215	88,142 (31.1)	195,073(68.9)	Reference	Reference	Reference
Female	238,875	84,314 (35.3)	154,561(64.7)	**1.303 (1.287, 1.319)**	**1.303 (1.287, 1.319)**	**1.303 (1.287, 1.319)**
Years of education completed, mean (SD)	4.6 (2.7)	5.4 (2.7)	4.2(2.6)	**1.225 (1.220, 1.229)**	**1.225 (1.220, 1.229)**	**1.224 (1.220, 1.229)**
Area-level socioeconomic status, n (%)						
Middle	184,565	41,841 (22.7)	142,724 (77.3)	Reference	Reference	Reference
Low	148,853	39,251 (26.4)	109,602 (73.6)	**1.241 (1.165, 1.323)**	**1.249 (1.172, 1.331)**	**1.254 (1.177, 1.336)**
High	113,223	31,622 (27.9)	81,601 (72.1)	**1.162 (1.008, 1.339)**	**1.163 (1.004, 1.348)**	**1.159 (1.000, 1.342)**
Geographic area, n (%)						
Suburban	168,780	39,515 (23.4)	129,265 (76.6)	Reference	Reference	Reference
Rural	147,104	37,759 (25.7)	109,345 (74.3)	0.981 (0.918, 1.048)	0.984 (0.921, 1.051)	0.993 (0.929, 1.062)
Urban	130,757	35,440 (27.1)	95,317 (72.9)	0.993 (0.865, 1.139)	0.986 (0.855, 1.137)	0.984 (0.854, 1.135)

Bold: $P < 0.05$.

Table S3.3: The estimated regression coefficients of changes in uncorrected visual acuity for both eyes among 990,578 students according to green space exposure in different buffers.

Factors	Model 1 NDVI$_{250m}$ included	Model 2 NDVI$_{500m}$ included	Model 3 NDVI$_{1000m}$ included
Normalized difference vegetation index (NDVI)	0.002 (-0.002, 0.006)	0.003 (-0.001, 0.007)	**0.004 (0.000, 0.009)**
UCVA at the initial screening	**-0.308 (-0.309, -0.307)**	**-0.308 (-0.309, -0.307)**	**-0.308 (-0.309, -0.307)**
Area-level socioeconomic status			
Middle	reference	reference	reference
High	0.018 (-0.001, 0.038)	0.018 (-0.001, 0.038)	0.019 (-0.001, 0.038)
Low	**-0.008 (-0.017, 0.000)**	**-0.009 (-0.017, -0.001)**	**-0.009 (-0.018, -0.001)**
Years of education completed	**-0.013 (-0.013, -0.012)**	**-0.013 (-0.013, -0.012)**	**-0.013 (-0.013, -0.012)**
Sex			
Male	reference	reference	reference
Female	**-0.018 (-0.019, -0.017)**	**-0.018 (-0.019, -0.017)**	**-0.018 (-0.019, -0.017)**
Geographic area			
Suburban	reference	reference	reference
Urban	**0.032 (0.013, 0.051)**	**0.031 (0.013, 0.050)**	**0.031 (0.013, 0.050)**
Rural	**0.030 (0.011, 0.050)**	**0.029 (0.010, 0.049)**	**0.028 (0.009, 0.048)**

Bold: $P < 0.05$. UCVA: uncorrected distance visual acuity.

Table S3.4: The estimated regression coefficients of changes in sphere equivalent for both eyes among 990,578 students according to green space exposure in different buffers.

Factors	Model 1 $NDVI_{250m}$ included	Model 2 $NDVI_{500m}$ included	Model 3 $NDVI_{1000m}$ included
Normalized difference vegetation index (NDVI)	0.001 (-0.011, 0.014)	0.000 (-0.013, 0.013)	-0.002 (-0.016, 0.012)
SER at the initial screening	**-0.075 (-0.076, -0.074)**	**-0.075 (-0.076, -0.074)**	**-0.075 (-0.076, -0.074)**
Area-level socioeconomic status			
Middle	Reference	Reference	Reference
High	**-0.084 (-0.145, -0.022)**	**-0.084 (-0.146, -0.022)**	**-0.084 (-0.146, -0.023)**
Low	**-0.121 (-0.147, -0.094)**	**-0.120 (-0.147, -0.094)**	**-0.120 (-0.146, -0.093)**
Years of education completed	**-0.016 (-0.017, -0.015)**	**-0.016 (-0.017, -0.015)**	**-0.016 (-0.017, -0.015)**
Sex			
Male	Reference	Reference	Reference
Female	**-0.026 (-0.030, -0.023)**	**-0.026 (-0.030, -0.023)**	**-0.026 (-0.030, -0.023)**
Geographic area			
Suburban	Reference	Reference	Reference
Urban	0.012 (-0.048, 0.071)	0.012 (-0.048, 0.071)	0.012 (-0.047, 0.072)
Rural	0.047 (-0.015, 0.109)	0.048 (-0.014, 0.110)	0.049 (-0.013, 0.111)

Bold: $P < 0.05$. SER: spherical equivalent refraction.

S4. Supporting tables for the results of sensitivity analysis in this study.

In the sensitivity analysis (n = 29,971), 51.9% of these students were males (n=15,569) and 48.1% were females (n = 14,402). The prevalence of myopia in this subgroup was 53.2%. There was no statistically significant difference in the distribution of sex between the subgroup and the overall group ($P > 0.05$). The students in this subgroup were younger, with an average age of 10.4 ± 2.8 years, compared to the overall group ($P < 0.05$). This suggests that parents of younger students were more likely to complete the questionnaire. In addition, student volunteers from low SES areas showed unwillingness to complete the questionnaire. As a result, there was a smaller proportion of students from low SES areas (n = 2,215), accounting for 7.4% of the total in this subgroup.

For the correlation between SES, green space, and the prevalence of myopia, these findings remained robust across sensitivity analyses. Students living in low SES areas exhibited a higher prevalence of myopia compared to those residing in middle SES districts, with ORs ranging from 1.312 to 1.335. The ORs and 95% CIs for NDVI at the 250-meter, 500-meter, and 1000-meter buffer were 0.949 (95% CI=0.913,0.987), 0.924 (95% CI=0.887,0.964), and 0.948 (95% CI=0.906,0.991), respectively. Moreover, students who frequented green spaces more often demonstrated a lower prevalence of myopia, with ORs ranging from 0.921 to 0.922. These findings are summarized in Table S4.1.

During the follow-up period, a total of 21,251 participants were included. Among them, 10,652 students were non-myopic at the initial screening, and 2,881 (27%) were newly diagnosed with myopia. The sensitivity analysis confirmed the robustness of the findings regarding SES. However, no evidence suggested a relationship between green space and the incidence of new myopia in the subgroup (Table S4.2). Nevertheless, higher greenness was correlated with a slower decline in UCVA ($NDVI_{250m}$, $NDVI_{500m}$, and $NDVI_{1000m}$) and SER (only for $NDVI_{250m}$). Students living in low SES areas exhibited a more rapid decline in UCVA. In contrast, those residing in high SES areas demonstrated a slower decline in UCVA than other SES groups (Table S4.3). Students living in high SES areas also experienced a rapid decline in SER (Table S4.4).

This study consistently found an association between parental myopia and poorer myopia outcomes in all sensitivity analyses. On a positive note, maintaining a reading distance of less than one foot from a book benefitted myopia outcomes. However, spending more time using screens was correlated with a faster decline in SER (Table S4.4). Moreover, students who frequently used desk lamps, such as LED, incandescent, or fluorescent lamps, while doing homework had a higher prevalence of myopia and experienced a rapid decline in UCVA compared to those who rarely used desk lamps.

Table S4.1: Results of the sensitivity analysis of factors associated with myopia among 29, 971 students in the TCARE study.

Table S4.2: Odds ratios (ORs) of new myopia among 10,652 students associated with socio-economic statuses and green space exposure.

Table S4.3: The estimated regression coefficients of changes in uncorrected visual acuity for both eyes among 21,251 students according to green space exposure in different buffers.

Table S4.4: The estimated regression coefficients of changes in sphere equivalent for both eyes among 21,251 students according to green space exposure in different buffers.

Table S4.1: Results of the sensitivity analysis of factors associated with myopia among 29,971 students in the TCARE study.

Factors	Myopia (n = 15,948)	Non-myopia (n = 14,023)	Model 1 $NDVI_{250m}$ included	Model 2 $NDVI_{500m}$ included	Model 3 $NDVI_{1000m}$ included
Constant	——	——	0.117	0.126	0.118
Normalized difference vegetation index (NDVI), P50 (P25, P75)	0.227 (0.200, 0.279)	0.233 (0.206, 0.281)	**0.949 (0.913, 0.987)**	**0.924 (0.887, 0.964)**	**0.948 (0.906, 0.991)**
Sex, n (%)					
Male	7,921 (49.7)	7,648 (54.5)	Reference	Reference	Reference
Female	8,027 (50.3)	6,375 (45.5)	**1.243 (1.181, 1.309)**	**1.244 (1.181, 1.309)**	**1.243 (1.181, 1.309)**
Years of education completed, P50 (P25, P75)	4.9 (2.9, 6.9)	2.9 (0.9, 4.9)	**1.508 (1.491, 1.525)**	**1.509 (1.492, 1.526)**	**1.509 (1.491, 1.526)**
Area-level socioeconomic status, n (%)					
Middle	7,721 (52.7)	6,921 (47.3)	Reference	Reference	Reference
Low	1,252 (56.5)	963 (43.5)	**1.312 (1.180, 1.459)**	**1.335 (1.200, 1.484)**	**1.312 (1.179, 1.460)**
High	6,975 (53.2)	6,139 (46.8)	1.051 (0.992, 1.114)	1.038 (0.979, 1.100)	1.050 (0.990, 1.113)
Parental myopia, n (%)					
Neither	5,108 (32.0)	5,534 (29.5)	Reference	Reference	Reference
Either	10,840 (68.0)	8,489 (60.5)	**1.915 (1.809, 2.027)**	**1.913 (1.807, 2.026)**	**1.914 (1.807, 2.026)**
Frequency of going to green spaces, n (%)					
≤1 per month	9,156 (57.4)	6,891 (49.1)	Reference	Reference	Reference
>2 per month	6,792 (42.6)	7,132 (50.9)	**0.921 (0.913, 0.987)**	**0.922 (0.875, 0.971)**	**0.921 (0.875, 0.971)**
Reading distance, n (%)					
Less than a foot	3,608 (22.6)	2,600 (18.5)	Reference	Reference	Reference
Greater than a foot	12,340 (77.4)	11,423 (81.5)	**0.800 (0.750, 0.853)**	**0.800 (0.750, 0.853)**	**0.800 (0.750, 0.853)**
Type of desk lamp, n (%)					
Hardly use a desk lamp	2,916 (18.3)	3,006 (21.4)	Reference	Reference	Reference
LED lights	9,160 (57.4)	7,883 (56.2)	**1.127 (1.040, 1.220)**	**1.124 (1.038, 1.217)**	**1.126 (1.039, 1.219)**
Others	3,872 (24.3)	3,134 (22.3)	**1.126 (1.050, 1.207)**	**1.123 (1.047, 2.204)**	**1.125 (1.049, 1.206)**

Bold: $P < 0.05$. Logistic regression models were used to determine the relationships between potential correlates and the prevalence of myopia. In the final step of regression, geographic area, outdoor play, screen time, sleeping with night lights, salt intake, snack and dessert intake, seafood intake, and soft drinks intake were removed from the model.

Table S4.2: Odds ratios (ORs) of new myopia among 10,652 students associated with socio-economic statuses and green space exposure.

Factors	Myopia (n = 2,881)	Non-myopia (n = 7,771)	Model 1 $NDVI_{250m}$ included	Model 2 $NDVI_{500m}$ included	Model 3 $NDVI_{1000m}$ included
Sex, n (%)					
Male	1,416 (24.7)	4,316 (75.3)	Reference	Reference	Reference
Female	1,465 (29.8)	3,455 (70.2)	**1.351 (1.238, 1.475)**	**1.351 (1.238, 1.475)**	**1.351 (1.238, 1.475)**
Years of education completed, P_{50} (P_{25}, P_{75})	3.9 (2.9, 4.9)	2.9 (2.8, 4.9)	**1.168 (1.142, 1.194)**	**1.168 (1.142, 1.194)**	**1.168 (1.142, 1.194)**
Area-level socioeconomic status, n (%)					
Middle	1,203 (21.5)	4,380 (78.5)	Reference	Reference	Reference
High	1,404 (33.5)	2,783 (66.5)	**1.695 (1.544, 1.860)**	**1.695 (1.544, 1.860)**	**1.695 (1.544, 1.860)**
Low	274 (31.1)	608 (68.9)	**1.623 (1.384, 1.904)**	**1.623 (1.384, 1.904)**	**1.623 (1.384, 1.904)**
Parental myopia, n (%)					
Neither	1,076 (23.7)	3,463 (76.3)	Reference	Reference	Reference
Either	1,805 (29.5)	4,308 (70.5)	**1.400 (1.275, 1.537)**	**1.400 (1.275, 1.537)**	**1.400 (1.275, 1.537)**
Reading distance, n (%)					
Less than a foot	605 (30.3)	1,389 (69.7)	Reference	Reference	Reference
Greater than a foot	2,276 (26.3)	6,382 (73.7)	**0.844 (0.756, 0.943)**	**0.844 (0.756, 0.943)**	**0.844 (0.756, 0.943)**
Sleeping with night lights, n (%)					
Never	2,521 (26.8)	6,889 (73.2)	Reference	Reference	Reference
Sometimes	360 (29.0)	882 (71.0)	1.128 (0.987, 1.289)	1.128 (0.987, 1.289)	1.128 (0.987, 1.289)

Bold: $P < 0.05$. Logistic regression models were used to determine the relationships between potential correlates and the prevalence of myopia. In the final step of regression, normalized difference vegetation index, geographic area, outdoor play, frequency of going to green spaces, screen time, type of desk lamp, salt intake, snack and dessert intake, seafood intake, and soft drinks intake were removed from the model.

Table S4.3: The estimated regression coefficients of changes in uncorrected visual acuity for both eyes among 21,251 students according to green space exposure in different buffers.

Factors	Model 1	Model 2	Model 3
	**$NDVI_{250m}$ included**	**$NDVI_{500m}$ included**	**$NDVI_{1000m}$ included**
Constant	**1.354 (1.300, 1.408)**	**1.351 (1.297, 1.405)**	**1.333 (1.279, 1.386)**
UCVA at the initial screening	**-0.271 (-0.281, -0.261)**	**-0.271 (-0.281, -0.261)**	**-0.271 (-0.281, -0.261)**
Sex			
Male	Reference	Reference	Reference
Female	**-0.020 (-0.026, -0.015)**	**-0.020 (-0.026, -0.015)**	**-0.021 (-0.026, -0.015)**
Years of education completed	**-0.013 (-0.014, -0.011)**	**-0.013 (-0.014, -0.011)**	**-0.013 (-0.014, -0.011)**
Low area-level socioeconomic status			
Middle and High	Reference	Reference	Reference
Low	**-0.027 (-0.038, -0.016)**	**-0.028 (-0.039, -0.017)**	**-0.028 (-0.039, -0.017)**
High area-level socioeconomic status			
Low and middle	Reference	Reference	Reference
High	**0.020 (0.014, 0.027)**	**0.021 (0.014, 0.027)**	**0.021 (0.014, 0.027)**
Normalized difference vegetation index (NDVI)	**0.004 (0.000, 0.009)**	**0.006 (0.021, 0.010)**	**0.009 (0.001, 0.015)**
Parental myopia			
Neither	Reference	Reference	Reference
Either	**-0.033 (-0.039, -0.027)**	**-0.032 (-0.038, -0.026)**	**-0.034 (-0.040, -0.028)**
Reading distance			
Less than a foot	Reference	Reference	Reference
Greater than a foot	**0.008 (0.001, 0.015)**	**0.008 (0.001, 0.015)**	**0.008 (0.001, 0.015)**
Type of desk lamp			
Hardly ever	Reference	Reference	Reference
LED lights	**-0.013 (-0.020, -0.005)**	**-0.013 (-0.020, -0.005)**	**-0.013 (-0.020, -0.005)**
Incandescent or fluorescent lamps	**-0.012 (-0.021, -0.004)**	**-0.012 (-0.021, -0.004)**	**-0.012 (-0.020, -0.003)**

Bold: $P < 0.05$. UCVA: uncorrected distance visual acuity. Logistic regression models were used to determine the relationships between potential correlates and the prevalence of myopia. In the final step of regression, geographic area, outdoor play, frequency of going to green spaces, screen time, sleeping with night lights, salt intake, snack and dessert intake, seafood intake, and soft drinks intake were removed from the model.

Table S4.4: The estimated regression coefficients of changes in sphere equivalent for both eyes among 21,251 students according to green space exposure in different buffers.

Factors	Model 1<br>$NDVI_{250m}$ included	Model 2<br>$NDVI_{500m}$ included	Model 3<br>$NDVI_{1000m}$ included
Constant	**-0.326 (-0.385, -0.266)**	**-0.273 (-0.308, -0.238)**	**-0.273 (-0.308, -0.238)**
SER at the initial screening	**-0.064 (-0.072, -0.057)**	**-0.065 (-0.072, -0.057)**	**-0.065 (-0.072, -0.057)**
Years of education completed	**-0.007 (-0.013, -0.001)**	**-0.008 (-0.013, -0.002)**	**-0.008 (-0.013, -0.002)**
Area-level socioeconomic status			
Low and middle	Reference	Reference	Reference
High	**-0.105 (-0.131, -0.078)**	**-0.111 (-0.135, -0.087)**	**-0.111 (-0.135, -0.087)**
Normalized difference vegetation index (NDVI)	**0.022 (0.005, 0.038)**	——	——
Parental myopia			
Neither	Reference	Reference	Reference
Either	**-0.091 (-0.116, -0.066)**	**-0.091 (-0.116, -0.067)**	**-0.091 (-0.116, -0.067)**
Screen time			
<2h	Reference	Reference	Reference
≧2h	**-0.035 (-0.060, -0.011)**	**-0.031 (-0.055, -0.007)**	**-0.031 (-0.055, -0.007)**

Bold: $P < 0.05$. SER: spherical equivalent refraction. Logistic regression models were used to determine the relationships between potential correlates and the prevalence of myopia. In the final step of regression, geographic area, outdoor play, frequency of going to green spaces, reading distance, type of desk lamp, sleeping with night lights, salt intake, snack and dessert intake, seafood intake, and soft drinks intake were removed from the model.

In model 2 and model 3, NDVI was also removed from the models.
